# NorA and Tet38 efflux pumps enable *Staphylococcus aureus* survival in the cystic fibrosis airway environment, resistance to antibiotics, and coinfection with *Pseudomonas aeruginosa*

**DOI:** 10.1128/aac.00460-25

**Published:** 2025-07-09

**Authors:** Q. C. Truong-Bolduc, Y. Wang, B. G. Lawton, J. J. Zweifach, G. M. McDevitt, Y. El Abdellaoui, H. Brown Harding, L. M. Yonker, L. G. Rahme, J. M. Vyas, D. C. Hooper

**Affiliations:** 1 Infectious Diseases Division and Medical Services, Massachusetts General Hospital, Harvard Medical School 1811 , Boston, Massachusetts, USA; 2 Department of Pediatrics, Massachusetts General Hospital, Harvard Medical School 1811 , Boston, Massachusetts, USA; 3 Department of Surgery, Massachusetts General Hospital, Harvard Medical School 1811 , Boston, Massachusetts, USA; The Peter Doherty Institute for Infection and Immunity, Melbourne, Victoria, Australia

**Keywords:** *S. aureus*, efflux pump, NorA, Tet38, *P. aeruginosa*, cystic fibrosis

## Abstract

Efflux pumps play multiple roles in bacterial physiology, environmental adaptation, and antibiotic resistance. Early cystic fibrosis (CF) airway infections start with *Staphylococcus aureus* (*SA*), often later followed by *Pseudomonas aeruginosa* (*PA*) infections. In this study, we have evaluated the role of *SA* pumps NorA and Tet38 in survival and interaction with *PA* in CF patients. Data showed a ≥4-log_10_CFU/mL growth deficit of *SA* mutants *ΔnorA* and *Δtet38* in an artificial sputum medium (ASM), suggesting NorA and Tet38 contributed to *SA* growth in CF sputum. In ASM, mucin activated *norA* but inhibited *tet38,* while extracellular DNA activated *tet38* but inhibited *norA*, demonstrating complementary roles of mucin and DNA in affecting NorA and Tet38 expression. Furthermore, exposure of *SA* wild type to *PA*-excreted molecules affected pump expression; 3,4-dihydroxy-2-heptylquinoline PQS caused an increase in *tet38* but a decrease in *norA*, 4-hydroxy-2-heptylquinoline HHQ caused a decrease in *norA* and *tet38*, and pyocyanin PYO caused a modest increase in *norA*, demonstrating differing additional roles of *PA*-secreted molecules influencing NorA and Tet38 expression. Evaluation of 48 randomly selected unique CF-associated *SA* showed that 18.8% were NorA-overexpressors and 10.4% were Tet38-overexpressors. NorA-overexpressors showed a fourfold increase in pyocyanin MIC and ≥16-fold in ciprofloxacin MIC. Furthermore, 89% of NorA-overexpressors carried an insertion of CAAT/ACAA/CTAT at the (−10) motif of the *norA* promoter, and 62.5% of these were co-isolated with *PA*.

These data showed that *SA* survives *PA* killing in CF sputum conditions using sputum key components and *PA*-specific signal molecules to regulate NorA and Tet38 expression.

## INTRODUCTION

*Staphylococcus aureus* is a common bacterium that can colonize human skin, nares, and gastrointestinal tract but also causes infections that range from minor skin infections to serious and life-threatening conditions, including endocarditis, osteomyelitis, and sepsis (1). The emergence of multidrug-resistant strains, such as methicillin-resistant *S. aureus* (MRSA), has created a healthcare challenge and complicated treatments to eradicate *S. aureus* infections. *Pseudomonas aeruginosa* is a ubiquitous environmental bacterium, commonly found in water, soil, and damp areas. Although usually harmless to healthy persons, *P. aeruginosa* can cause serious infections in individuals with chronic lung disease, such as persons with cystic fibrosis (pwCF), in whom *P. aeruginosa* can persist for an extended time following the initial infection (2).

Cystic fibrosis (CF) affects more than 100,000 children and adults worldwide with over 40,000 pwCF living in the United States (3). Impaired mucus clearance in pwCF creates a dehydrated and thickened mucus environment that contains elevated levels of mucin, extracellular DNA, and other components that form a habitat for opportunistic microbes such as *S. aureus* and *P. aeruginosa*, both of which are designated as ESKAPE pathogens, a group of six bacteria posing a significant threat to public health, and for which antibiotic resistance is a common problem (4, 5). In addition, these two pathogens are also isolated from infected wounds such as diabetic foot ulcers, burns, and surgical wounds (5–7).

Since *S. aureus* colonization of the CF airways often precedes colonization with *P. aeruginosa* (8), we performed experiments to address the responses of *S. aureus* to components of the CF sputum as well as to exotoxins secreted by *P. aeruginosa* during a coinfection. CF sputum is a complex habitat that affects the behaviors of bacteria that dwell in the CF airways (9–11). Despite advances in CFTR modulators (CF Transmembrane Conductance Regulator), many pwCF remain colonized with pathogenic bacteria and suffer from pulmonary exacerbations, pulmonary decline, and death due to these pulmonary infections. CFTR modulators improve but do not normalize airway hydration, nor can they restore CF-related bronchiectasis, and as a result, CF airways can remain coated with viscous mucus layers which may persistently harbor *S. aureus* and/or *P. aeruginosa* (12, 13). Artificial sputum media (ASM) have been developed to reflect the conditions of the CF airway. ASM media mimic the sputum of the CF airways, with mucin and extracellular DNA as two key components among other compounds (9, 14). ASM media have been used in several studies of *P. aeruginosa* cultured alone or together with *S. aureus* in *in vitro* assays (15).

Several studies of the interactions between *S. aureus* and *P. aeruginosa* demonstrated that a coinfection of *S. aureus* with *P. aeruginosa* enhances antibiotic resistance and virulence in both pathogens, with *P. aeruginosa* as the more aggressive member of the pair (16, 17). Recent studies demonstrated that while initially inhibited by *P. aeruginosa* secreted products, *S. aureus* quickly became resistant to these toxins (18, 19). The mechanism by which *S. aureus* protects itself against *P. aeruginosa* killing is not well understood. *S. aureus* can export a large variety of unrelated compounds, including antibiotics, due to its large collection of efflux pumps and transporters. These native membrane proteins confer in *S. aureus* a low level of resistance to their corresponding substrates by decreasing the intracellular concentration of antimicrobial compounds using their active efflux (20–22). Among reported *S. aureus* efflux pumps and transporters, NorA and Tet38 MDR efflux pumps (23) that confer a low-level resistance to fluoroquinolones (ciprofloxacin and levofloxacin) and tetracycline, respectively, have been shown to protect *S. aureus* against *P. aeruginosa* exoproducts as well as antibiotics. Recently, we demonstrated that NorA and Tet38 protect from pyocyanin (PYO) and phenazine-1 carboxylic acid (PCA) of *P. aeruginosa*, respectively (24, 25). PYO is a quorum-sensing (QS)-dependent virulence factor and is converted from PCA through the action of enzymes encoded by genes *phzS* and *phzM* (26, 27). PYO production, the *P. aeruginosa* QS signal molecule 3,4-dihydroxy-2-heptylquinoline (PQS), and its precursor 4-hydroxy-2-heptylquinoline (HHQ) (28, 29) require the function of the QS Multiple Virulence Factor Regulator (MvfR), also known as PqsR. Both PQS and HHQ act as ligands of *P. aeruginosa* QS regulator MvfR to modulate the expression of multiple QS-regulated genes encoding *P. aeruginosa* virulence factors such as PYO (29, 30). Other studies, however, highlighted the high levels of HHQ in the infected tissues of mice and humans and the dispensable role of PQS in *in vivo* virulence, underscoring a potential contribution of HHQ to *P. aeruginosa* virulence *in vivo* (29, 31).

In this study, we investigated the contribution of *S. aureus* NorA and Tet38 to adaptation to growth in conditions mimicking those of the CF airways in ASM and the effects of *P. aeruginosa* secreted signal molecules PQS, HHQ, and PYO on the expression of *S. aureus* efflux pumps NorA and Tet38. We demonstrated that in ASM, mucin induced NorA but inhibited Tet38 expression, while extracellular DNA induced Tet38 but inhibited NorA expression. We also showed that PQS repressed NorA expression but induced Tet38, HHQ repressed both NorA and Tet38, and PYO slightly induced NorA but not Tet38. Furthermore, we demonstrated that NorA and Tet38 both contributed to the growth of *S. aureus* in ASM, with NorA playing the larger role, a finding possibly underlying the frequency of *S. aureus* isolates from pwCF with mutations associated with an increase in NorA expression. Our data also suggested that *P. aeruginosa* could inhibit *S. aureus* growth by limiting the expression of efflux pumps necessary for *S. aureus* growth in the environment of CF sputum.

## RESULTS

### *S. aureus* requires functional NorA and Tet38 efflux pumps to grow in ASM medium

We examined the roles of NorA and Tet38 in *S. aureus* (*SA*) survival in ASM medium by performing growth curve assays using RN6390 as the *SA* reference strain (WT) (32), and the *SA* efflux pump mutants *ΔnorA, Δtet38*, and *ΔnorC* (24, 32, 33). We included the pump mutant *ΔnorC* (33) as a control of the efflux pump that was unaffected when *SA* was grown in ASM media. *SA* NCTC8325-4 is the parental strain of *ΔnorA* (SA-K1758) (34), and RN6390 is the parental strain of mutants *Δtet38* and *ΔnorC* (32, 33). RN6390 is a derivative of NCTC8325-4, and both parental strains showed similar growth curves in ASM (24).

We started fresh cultures of *SA* WT, pump mutants, and NorA- and Tet38-complementing strains in ASM from overnight cultures in TSB (initial CFU/mL ~ 10^4^).

**Fig 1:**
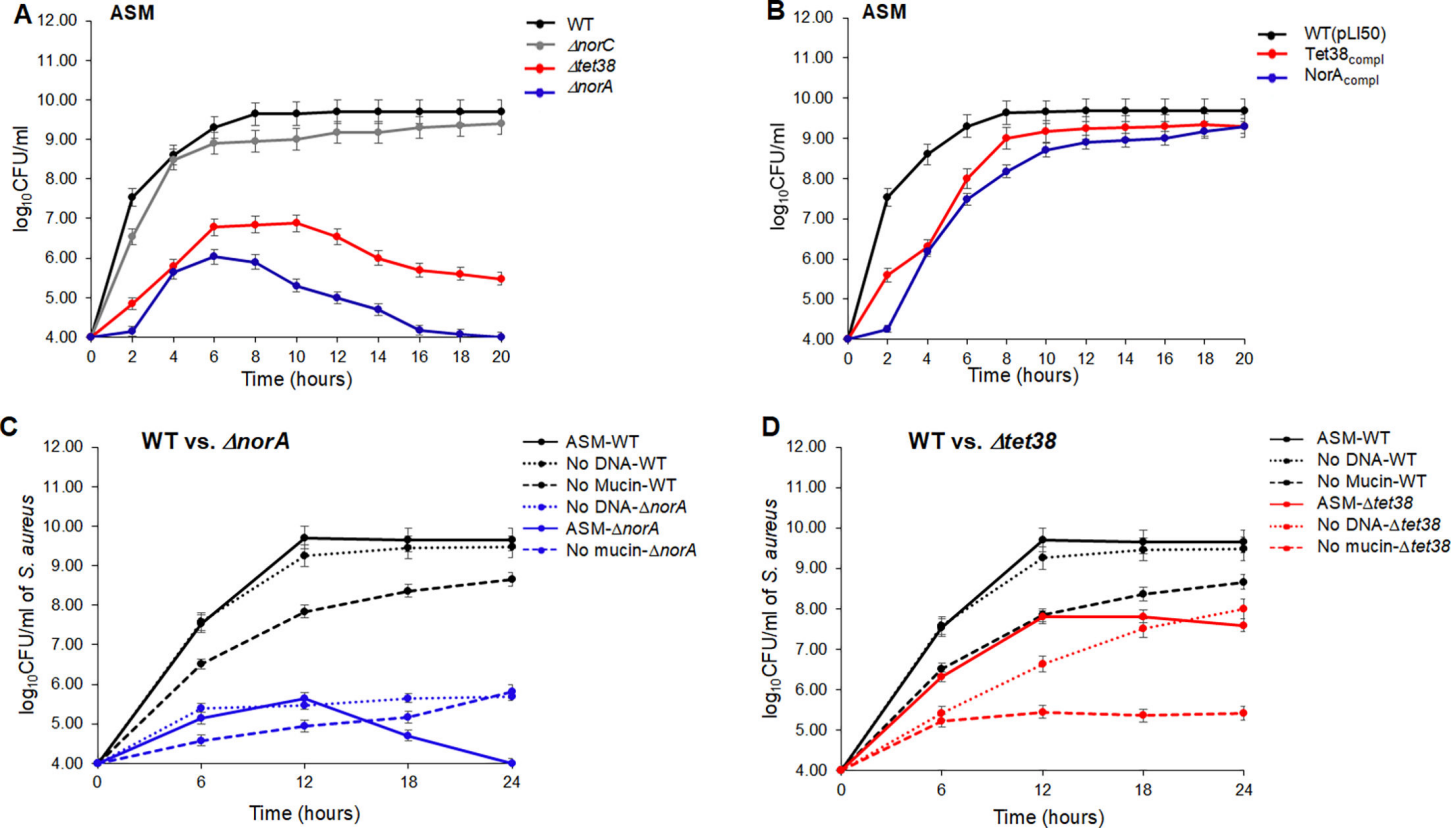
*S. aureus* WT and pump mutant growth curves in various ASM media. *S. aureus* growth curves in ASM medium. WT, efflux pump mutants (**A**), and mutant strains complemented with the corresponding pump genes (**B**) were cultured in ASM medium for 20 hours, and then plating and colony counting were performed. The growth curve assays were repeated three times with three different biological samples. The error bars represent the means of (log_10_CFU/mL ± SD) for each assay. The differences in the log_10_CFU/mL of mutants *ΔnorA* and *Δtet38* compared to that of WT and mutant *ΔnorC* were statistically significant as determined by a one-way ANOVA (*P < 0.05*). WT, *S. aureus* RN6390; WT(pLI50), RN6390 transformed with the empty plasmid. NorA_compl_, mutant *ΔnorA* transformed with plasmid construct (pLI50-*norA*). Tet38_compl_, mutant *Δtet38* transformed with plasmid construct (pLI50-*tet38*). *S. aureus* growth curves in ASM, ASM without mucin, and ASM without DNA. WT, efflux pump mutant *ΔnorA* (**C**), or *Δtet38* (**D**) were cultured in ASM medium, ASM without 0.5% of mucin or 0.4% of DNA for 20 hours, and then colony counting was performed. The growth curve assays were repeated three times with three different biological samples. The error bars represent the means of (log_10_CFU/mL ± SD) for each assay. The differences in the log_10_CFU/mL of mutant *ΔnorA* or *Δtet38* compared to that of WT in three ASM media were statistically significant as determined by a one-way ANOVA (*P < 0.05*). The differences in the log_10_CFU/mL between *Δtet38* in ASM and ASM without DNA vs. ASM without mucin was statistically significant as determined by a one-way ANOVA (*P <* 0.05). WT, *S. aureus* RN6390; *ΔnorA*, mutant lacks NorA but expresses Tet38; *Δtet38*, mutant lacks Tet38 but expresses NorA. ASM, medium prepared with mucin and DNA (14). No DNA, ASM medium prepared without 0.4% of fish sperm DNA; No mucin, ASM medium prepared without 0.5% of mucin from porcine stomach. WT vs. *ΔnorA*, *norA* mutant compared to WT; WT vs. *Δtet38*, *tet38* mutant compared to WT.

After 20 hours of growth, we found that *∆norA* and *∆tet38* mutants exhibited a growth deficit of 6-log_10_CFU/mL and 4-log_10_CFU/mL, respectively, when compared to the growth of *SA* WT and *∆norC* in ASM (Fig. 1A). These growth deficits were restored when NorA- and Tet38-complementing strains were cultured under the same conditions (Fig. 1B). Mutant *∆norA,* mutant *∆tet38*, RN6390, and NCTC8325-4 had the same growth rates in TSB.

These data showed that functional NorA and Tet38 were both important for *S. aureus* to grow in ASM, mimicking CF sputum conditions. Notably, a lack of NorA was more damaging to *SA* survival than a lack of Tet38, as the *∆norA* mutant showed a larger growth deficit compared to that of the *∆tet38* mutant after 20 hours in ASM medium (2− log_10_CFU/mL difference) (Fig. 1A).

To confirm that these effects were not specific to the laboratory strains we used, we also tested strain USA300 and its *norA* (SAUSA300_0680) and *tet38* (SAUSA300_0139) mutants from the NARSA Nebraska Transposon Library (35). Growth curve assays were performed in ASM medium for 20 hours at 37 °C, and we found that USA300 *norA* and *tet38* mutants also showed a growth deficit of 5 − log_10_CFU/mL and 4 − log_10_CFU/mL, respectively.

### Mucin and DNA of the ASM medium have opposite effects on *norA* and *tet38* expression

We grew *SA* WT (RN6390), *∆norA*, and *∆tet38* mutants in ASM, ASM without 0.5% porcine stomach mucin (wt/vol), and ASM without 0.4% fish sperm DNA (wt/vol) based on the composition of the ASM medium (14) where mucin and DNA are both components. *SA* (10^4^ CFU/mL) were cultured at 37 °C for 24 hours under shaking. Compared to WT, *∆norA* mutant showed a growth deficit of 6 − log_10_CFU/mL in ASM but grew slightly better in ASM without mucin (deficit of 3 − log_10_CFU/mL) or without extracellular DNA (deficit of 4 − log_10_CFU/mL) (Fig. 1C). Compared to WT, *∆tet38* mutant showed a similar growth deficit in ASM or ASM without DNA (deficit of ~2.5 − log_10_CFU/mL). In ASM without mucin, *∆tet38* mutant showed a growth deficit of 3.5 − log_10_CFU/mL for 18 hours (6–24 hours) when compared to that of WT (Fig. 1D). These data suggested that NorA and Tet38 individually played significant roles in *S. aureus* growth in CF sputum, with NorA apparently serving a greater role in bacterial survival in this environment.

We carried out real-time qPCR assays to compare *norA* and *tet38* transcript levels in *SA* WT, *∆norA* mutant, and *∆tet38* mutant when grown in ASM, ASM without mucin, and ASM without DNA to those of bacteria grown in TSB. The housekeeping gene *gmk* was used as an internal control. The *gmk* transcript level remained unchanged whether *S. aureus* was cultured in TSB or ASM with or without mucin or DNA (Table S1). The *norA* and *tet38* transcript levels were normalized to those of *S. aureus* grown in TSB.

**Fig 2:**
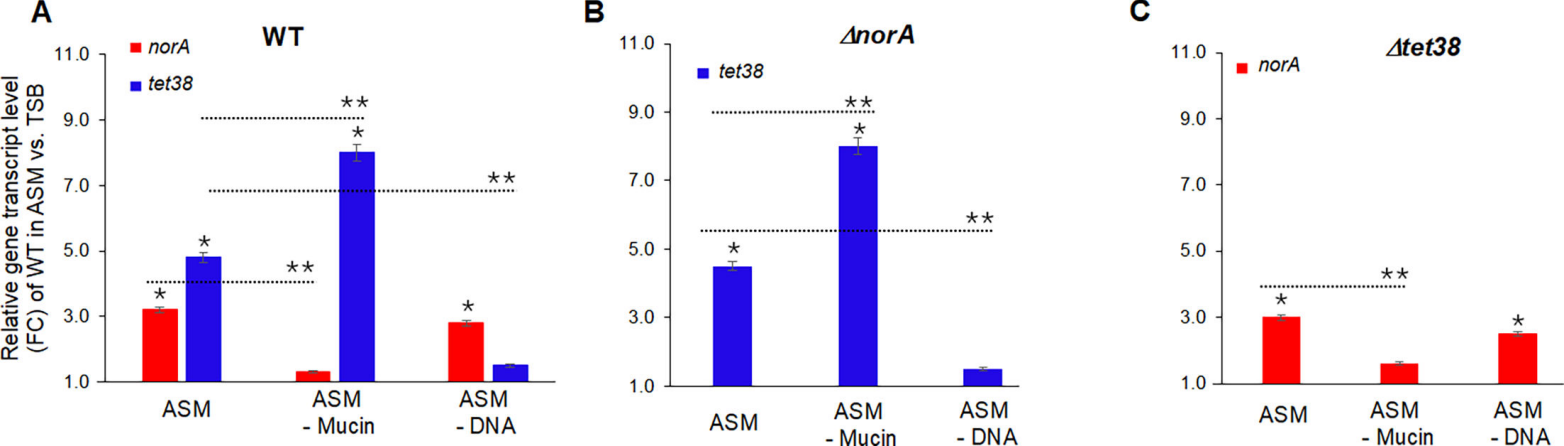
Gene transcript levels in ASM media and ASM media without mucin or DNA. *S. aureus* WT (**A**), mutant *ΔnorA* (**B**), and mutant *Δtet38* (**C**) were cultured in TSB, ASM, ASM without mucin (ASM – Mucin), and ASM without DNA (ASM – DNA) (initial CFU/mL ~ 10^4^) for 1 hour, then quantitative real-time RT-PCR assays (qPCR) were performed to assess the level of *norA* and *tet38* of WT (**A**), *tet38* of mutant *ΔnorA* (**B**), and *norA* of mutant *Δtet38* (**C**). The relative transcript levels of *norA* and *tet38* were expressed as the fold change (FC) in pump gene transcripts of bacteria grown in ASM, ASM without mucin, or ASM without DNA versus bacteria grown in TSB. The assays were repeated three times with three different biological samples. The error bars represent the means of FC ± SEM for each assay. The (*) represents the differences in the FC of *norA* or *tet38* of WT or mutants grown in various ASM versus TSB; and (**) represents the differences in the FC of *norA* or *tet38* of WT or mutants grown in ASM without mucin or DNA versus ASM. The differences were statistically significant as determined by an ANOVA (*P <* 0.05).

In ASM medium and compared to TSB, *norA* and *tet38* transcripts of WT increased threefold and fivefold, respectively. In ASM without mucin and compared to TSB, *norA* remained unchanged (fold change [FC] of *norA* in TSB ~ 1.0), and *tet38* increased eightfold. In ASM without mucin and compared to ASM, *norA* decreased threefold, and *tet38* increased ~twofold. In ASM without DNA and compared to TSB, *norA* increased threefold, and *tet38* remained unchanged. In ASM without DNA and compared to ASM, *norA* remained unchanged, and *tet38* decreased fourfold (Fig. 2A). These data showed that mucin induced *norA* expression but repressed *tet38* expression, while DNA induced *tet38* but repressed *norA*, establishing complementary responses to these two key components of ASM.

The *tet38* transcript levels of mutant *∆norA* and the *norA* transcript levels of mutant *∆tet38* in ASM, ASM without mucin, or ASM without DNA were similar to those of *SA* WT grown in the same ASM medium (Fig. 2B and C). Therefore, relative to *SA* WT grown in different versions of ASM media, induction of *tet38* in response to mutant *∆norA* or induction of *norA* in response to mutant *∆tet38* was unaffected by the absence of NorA or Tet38, respectively. By contrast, despite an overexpression of Tet38, mutant *∆norA* grew poorly in ASM and ASM without mucin or DNA (Fig. 1C; Fig. 2A and B). These findings showed that, in the absence of NorA, Tet38 could only ensure a minimum growth of the mutant *∆norA.* Thus, NorA was particularly important for *SA* WT growth in ASM (CF sputum by proxy), and the data suggested that Tet38 might be involved in other *SA* physiological functions.

Notably, in the absence of Tet38, mutant *∆tet38* grew relatively well in ASM without DNA and showed less growth deficit in ASM (Fig. 1D). In ASM without mucin, mutant *∆tet38* showed a stagnant growth curve similar to that of mutant *∆norA* (Fig. 1C and D). These findings showed that NorA alone could compensate for the lack of Tet38 and allowed mutant *∆tet38* to grow in ASM with or without DNA. In ASM without mucin, a low level of NorA expression due to a lack of mucin (no induction of *norA*) and the presence of DNA (repression of *norA*) led to a reduction in the growth of mutant *∆tet38* (Fig. 1D; Fig. 2A and C).

Taken together, findings from mutants *∆norA* and *∆tet38* showed that the induction of *norA* and *tet38* by different ASM media was independent of each other. These results also showed that the survival of *S. aureus* in a CF airway-type environment is supported by overexpression of NorA and/or Tet38 and that the mucin and DNA components of this environment support their expression in a complementary manner.

We carried out a growth curve assay using the CF strain CF-1 (Tables 1 and 2) that overexpressed both *norA* (4.3-fold increase) and *tet38* (3.2-fold increase) in ASM and ASM without mucin. The CF-1 strain grew similarly to the WT strain in both ASM media. This suggested that mucin was important as a nutrient source in ASM (data not shown).

**TABLE 1:** CF-associated *S. aureus* (CF-SA) clinical strains and *S. aureus* transformants used in this study^*a*^^,^^*e*^

					MIC (µg/mL)			
CF-SA #^*g*^	Characteristics^*b*^		CFTR modulator	norA promoter	CIP		PYO	TET
Newman	MSSA		–^*f*^	Wild type	0.5		6	0.5
CF-*SA* with wild-type *norA* and *tet38* promoters:								
CF-1	MSSA		None	Wild type	**8**		12	2
CF-2	MSSA		None	Wild type	0.5		6	0.5
CF-3	MSSA		None	Wild type	0.5		6	0.5
CF-4	MSSA		None	Wild type	0.5		6	0.5
CF-5	MSSA		ETI	Wild type	0.5		6	2
CF-6	MSSA		ETI	Wild type	0.5		6	0.5
CF-7	MRSA		Ivacaftor	Wild type	0.5		6	0.5
CF-8	MSSA		ETI	Wild type	0.5		6	0.5
CF-9	MSSA		ETI	Wild type	0.5		6	0.5
CF-10	MSSA		VTD	Wild type	0.5		6	0.5
CF-11	MSSA		None	Wild type	0.5		6	0.5
CF-12	MSSA/PA		ETI	Wild type	0.5		6	0.5
CF-13	MSSA/PA		ETI	Wild type	0.5		6	0.5
CF-14	MSSA/PA		ETI	Wild type	0.5		6	0.5
CF-15	MSSA/PA		LI, ETI	Wild type	0.5		6	0.5
CF-16	MSSA		ETI	Wild type	1		6	0.5
CF-*SA* with wild-type *tet38* promoter and *norA* promoter with a polX insertion^*c*^ :								
CF-17	MSSA		ETI	341bp-*polX*	0.5		3	0.5
CF-18	MRSA		ETI	341bp-*polX*	0.5		3	0.5
CF-19	MRSA		None	341bp-*polX*	0.5		3	0.5
CF-20	MSSA		None	341bp-*polX*	0.5		3	0.5
CF-21	MSSA		ETI	341bp-*polX*	0.5		3	0.5
CF-22	MSSA/PA		ETI	341bp-*polX*	0.5		3	0.5
CF-*SA* with wild-type *tet38* promoter and norA promoter with a 4-nucleotide insertion^*d*^ :								
CF-23	MSSA/PA		Vanzacafor/ivacaftor	CAAT	**16**		24	2
CF-24	MSSA		None	CAAT	**128**		24	2
CF-26	MSSA		ETI	CAAT	**16**		12	0.5
CF-27	MRSA/PA		ETI	ACAA	**>128**		24	0.6
CF-28	MRSA/PA		ETI	CAAT	**32**		24	0.6
CF-29	MRSA/PA		ETI	CAAT	**128**		24	0.6
CF-30	MRSA/PA		None	CTAT	**128**		24	2
*S. aureus* RN6390 (WT) overexpressing NorA from plasmid constructs:								
WT (pLI50)				Empty plasmid	0.5		6	0.5
WT [pLI50-*norA*_(WT)_p-*norA*]				*norA* with wild-type promoter	1		12	0.5
WT [pLI50-*norA*_(CAAT)_p-*norA*]				*norA* with CAAT insertion	2		24	0.5
WT [pLI50-*norA*_(ACAA)_p-*norA*]				*norA* with ACAA insertion	2		18	0.5
WT [pLI50-*norA*_(CTAT)_p-*norA*]				*norA* with CTAT insertion	2		16	0.5
*norA* mutant (∆*norA*) overexpressing NorA from plasmid constructs:								
∆*norA* (pLI50)				Empty plasmid	0.25		3	0.5
∆*norA* [pLI50-*norA*_(WT)_p-*norA*]				*norA* with wild-type promoter	0.5		6	0.5
∆*norA* [pLI50-*norA*_(CAAT)_p-*norA*]				*norA* with CAAT insertion	2		24	0.5
∆*norA* [pLI50-*norA*_(ACAA)_p-*norA*]				*norA* with ACAA insertion	2		18	0.5
∆*norA* [pLI50-*norA*_(CTAT)_p-*norA*]				*norA* with CTAT insertion	2		18	0.5

^*a*^ CF, cystic fibrosis; CIP, ciprofloxacin; PYO, pyocyanin; TET, tetracycline; ETI, elexacaftor/tezacaftor/ivacaftor (Trikafta); LI, lumacaftor/ivacaftor (Orkambi), VTD, vanzacaftor, tezacaftor, deutivacaftor (Alyftrek).

^*b*^ *S. aureus* isolated alone or co-isolated with *P. aeruginosa*. All CF-SA strains were collected from patients' sputum or throat samples. CF-25 and CF-26: CF-SA isolated from pwCF previously colonized with *P. aeruginosa* (2010 and 2011).

^*c*^ *polX*, gene encoding *S. aureus* DNA polymerase. CF-SA strains carrying an insertion of 341 bp of the polX gene at the (−10) consensus sequence of the *norA* promoter.

^*d*^ CF-SA strains carrying an insertion of a 4-nucleotide CAAT, ACAA, or CTAT at the (−10) consensus sequence of the *norA* promoter.

^*e*^ Values in boldface represent CF-*SA* with an increase in the MICs of ciprofloxacin 16-fold or more compared to the MIC of Newman. *norA*p-*norA*, *norA* promoter fused to *norA* gene; *norA*(CAAT)p*-norA*, *norA* promoter with CAAT insertion fused to *norA* gene; *norA*(ACAA)p-*norA*, *norA* promoter with ACAA insertion fused to *norA* gene; *norA*(CTAT)p-*norA*, *norA* promoter with CTAT insertion fused to *norA* gene. WT, RN6390; ∆*norA*, *norA* mutant.

^*f*^ – indicates the numbering of the CF strain of the study.

^*g*^ # indicates the number of *S. aureus*.

**TABLE 2:** Transcript levels of norA and tet38 of CF-SA cultured in TSB

*S. aureus*	Relative transcript level (FC) of efflux pumps (mean FC ± SD)^*a*^	
	norA	tet38
WT	1	1
CF-*SA* with wild-type *norA* promoter:		
**CF-1**	**4.3** ± 0.01	3.2 ± 0.1
CF-2	1.3 ± 0.02	1.2 ± 0.01
CF-3	0.95 ± 0.05	0.9 ± 0.01
CF-4	1.02 ± 0.01	1.05 ± 0.02
**CF-5**	0.98 ± 0.05	**3.8** ± 0.03
CF-6–CF-16^*b*^	0.8 ± 0.01–1.2 ± 0.02	0.5 ± 0.01–1.5 ± 0.02
CF-*SA* with *polX* insertion in the *norA* promoter:		
CF-17–CF-22^*b*^	0.2 ± 0.05–0.5 ± 0.03	0.9 ± 0.01–1.7 ± 0.05
CF-*SA* with CAAT, ACAA, or CTAT insertion in the *norA* promoter:		
**CF-23**	**8.5** ± 0.5	**3.0** ± 0.05
**CF-24**	**15.4** ± 0.5	**3.02** ± 0.05
**CF-30**	**10.9** ± 0.5	**2.5** ± 0.02
**CF-25–CF-29** ^*b*^	**6.0** ± 0.2**–15.4** ± 0.3	0.74 ± 0.02–1.5 ± 0.05

^*a*^ Relative gene expression determined as the fold change (FC) of *norA* or *tet38* gene transcripts of CF-*SA* strains cultured in TSB compared to that of the reference strain Newman (WT). The gmk gene was used as an internal control. Each assay was done in triplicate, and RNAs were collected from three independent biological samples. All values represent the means of three independent experiments. Values in boldface represent differences in *norA* or *tet38* transcripts between CF-*SA* and WT that are statistically significant as determined by a one-way ANOVA (*P* < 0.05).

^*b*^ CF-*SA* strains with similar FC of *norA* or *tet38* transcript levels.

**TABLE 3:** Bacterial strains, plasmids, and primers used in this study

Strains, plasmids, and primers	Genotypes or relevant characteristic(s)	Reference or source
*S. aureus*		
RN6390	Wild type	(32)
NCTC-8325-4	Derivative of NCTC8325	(36)
Newman	Laboratory strain, high level of clumping factor	(37)
Δ*norA*	*norA* mutant (SA-K1758) from NCTC8325-4	(24, 34)
Δ*tet38*	Δ*tet38* (YW22) deletion mutant from RN6390	(32)
Δ*norC*	Δ*norC* deletion mutant from RN6390	(33)
RN6390 or Δ*norA* (pLI50)	Empty plasmid, Cm^R^	(24)
RN6390 (pLI50-*norA*)	NorA-overexpressor, Cm^R^	(24)
RN6390 (pLI50-*tet38*)	Tet38-overexpressor, Cm^R^	(25)
Δ*norA* (pLI50-*norA*)	NorA-complementing strain, Cm^R^	This study
Δ*tet38* (pLI50-*tet38*)	Tet38-complementing strain, Cm^R^	This study
RN6390 [pLI50-*norA*_(WT)_p-*norA*]	*norA* with wild-type promoter, Cm^R^	This study
RN6390 [pLI50-*norA*_(CAAT)_p-*norA*]	*norA* with mutated promoter, Cm^R^	This study
RN6390 [pLI50-*norA*_(ACAA)_p-*norA*]	*norA* with mutated promoter, Cm^R^	This study
RN6390 [pLI50-*norA*_(CTAT)_p-*norA*]	*norA* with mutated promoter, Cm^R^	This study
Δ*norA* [pLI50-*norA*_(WT)_p-*norA*]	*norA* with wild-type promoter, Cm^R^	This study
Δ*norA* [pLI50-*norA*_(CAAT)_p-*norA*]	*norA* with mutated promoter, Cm^R^	This study
Δ*norA* [pLI50-*norA*_(ACAA)_p-*norA*]	*norA* with mutated promoter, Cm^R^	This study
Δ*norA* [pLI50-*norA*_(CTAT)_p-*norA*]	*norA* with mutated promoter, Cm^R^	This study
CF-associated *S. aureus* (CF-SA)		
CF-1 – CF30	*S. aureus* clinical strains isolated from pwCF	This study
*P. aeruginosa*		
PA14	Reference strain, virulent burn wound isolate	(38)
PA-12	Co-isolated with CF-SA CF-12	This study
PA-13	Co-isolated with CF-*SA* CF-13	This study
PA-14	Co-isolated with CF-*SA* CF-14	This study
PA-15	Co-isolated with CF-*SA* CF-15	This study
*E. coli*		
DH5α	F^-^ Φ80*lac*ZΔM15 Δ(*lac*ZYA-*arg*F) U169 *rec*A1 *end*A1	Life Technologies
	*hsd*R17(r_k_^-^, m_k_^+^) *pho*A *sup*E44 *thi*-1 *gyr*A96 *rel*A1 λ^-^	
Plasmid		
pLI50	Shuttle plasmid *E. coli*–*S. aureus*, Cm^R^	(32)
Primers for real-time RT-PCR assays:		
*gmk*	Forward 5′ TCAGGACCATCTGGAGTAGGTAAAG 3′	
	Reverse 3′ CAAATGCGTGAAGGTGAAGTTGATG 5′	
*norA*	Forward 5′ TGGCCACAATTTTTCGGTAT 3′	
	Reverse 3′ CTTTGGCTACATGTCAGCGA 5′	
*tet38*	Forward 5′ ATCGTAGTATTTACGTTGCC 3′	
	Reverse 3′ GGCTTAATTCTAGTGGCAAC 5′	
Primers for *norA* overexpressor with a wild-type 359-bp *norA* promoter:		
*norA*-PF (BamHI)	Forward 5′ TGCGGATCCATTTTAATACAACG 3′	
*norA*-(EcoRI)-R	Reverse 5′ ATGCGAATTCCATATTTTGTTCTT 3′	
Primers for plasmid constructs carrying a mutated norA promoter with a 4-nucleotide insertion:		
*norA*-P-F1	Forward 5′ ATGTTGTAATACAAACAATATAGAAACTTTTT 3′	
*norA*-P-F2	Forward 5′ ATGTTGTAATACAATCAATATAGAAACTTTTT 3′	
*norA*-P-F3	Forward 5′ ATGTTGTAATACAATCTATATAGAAACTTTTT 3′	
*norA*-P-R	Reverse 5′ TGCTACATTTGACAATATTTTTTCTTTGTAAT 3′	

### *P. aeruginosa* exoproducts also affect expression of Tet38 and NorA

To evaluate the influence of *P. aeruginosa* (*PA*)-secreted molecules on the transcript levels of *SA norA* and *tet38*, we exposed RN6390 (*SA* WT; 10^5^ CFU/mL) to growth supernatants of *PA* reference strain PA14 (*PA* WT) (38) that was prepared from an overnight culture in TSB (30% of *PA* WT supernatant + 70% of TSB) as previously described (18). *SA* WT was also added in parallel in the control assay medium (30% saline +7 0% of TSB) for comparison. The *SA* WT/*PA* supernatant ggrewat 37 °C under shaking for 1 hour.

**Fig 3:**
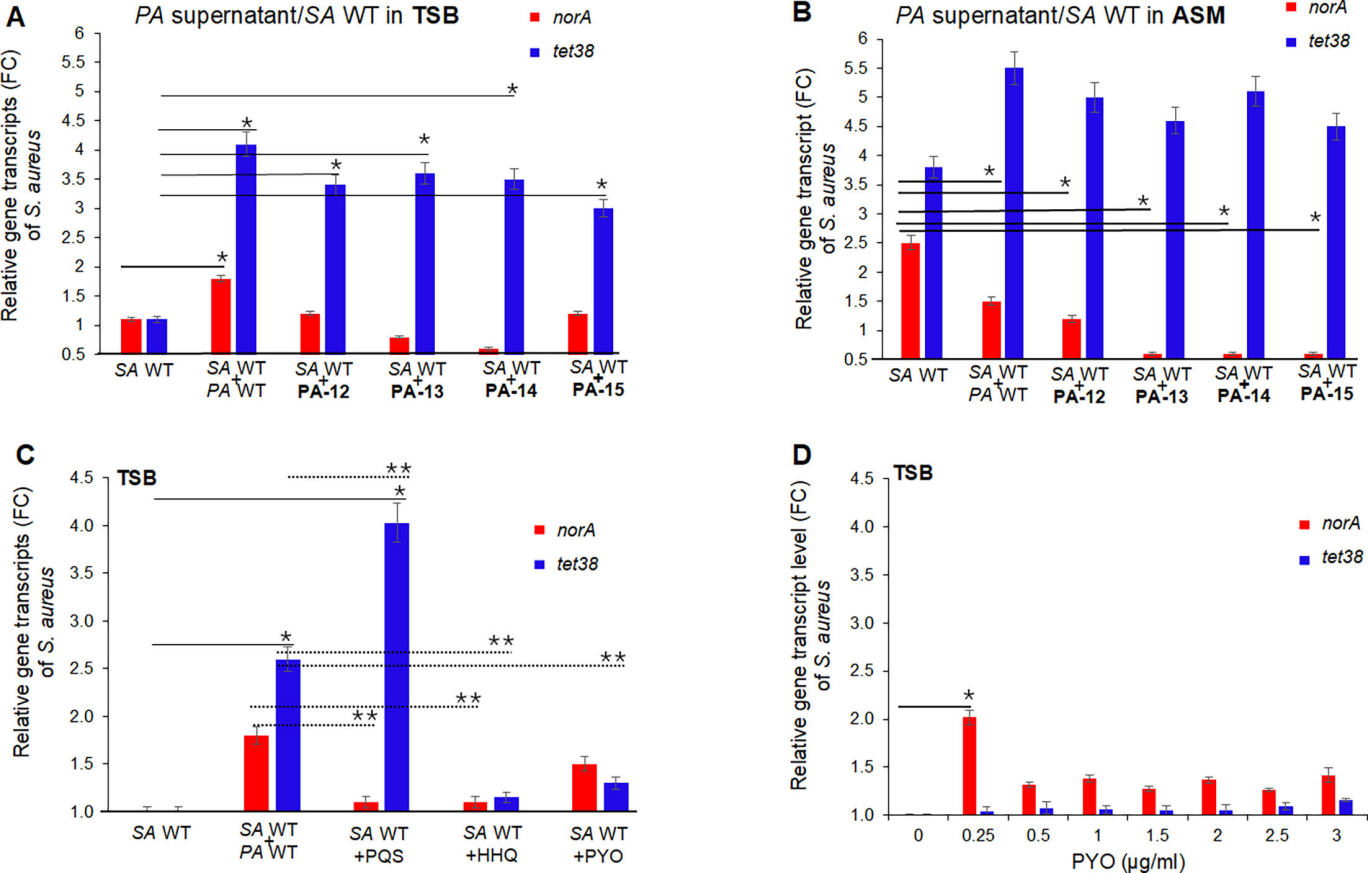
Variation of *SA norA* and *tet38* transcripts in different conditions. *norA* and *tet38* transcripts of *SA* WT exposed to supernatants of *PA*. The *PA* supernatants were prepared from overnight cultures in TSB. *SA* WT grew in TSB or ASM until OD_600_ ~0.5, then 30% *PA* supernatant was added for 1 hour. Quantitative real-time RT-PCR assays (qPCR) were performed to assess the level of *norA* and *tet38* of WT exposed to *PA* in TSB (**A**) or WT exposed to *PA* in ASM (**B**). The relative transcript levels of *norA* and *tet38* were expressed as the fold change (FC) in pump gene transcripts of *SA* WT grown in TSB or ASM and exposed to *PA* supernatants versus non-exposed *SA* WT. The assays were repeated three times with three different biological samples. The error bars represent the means of FC ± SEM for each assay. The (*) represents the differences in the FC of *norA* or *tet38* of *SA* WT in TSB or ASM and exposed to *PA* supernatants versus *SA* WT non-exposed in TSB or ASM. The differences were statistically significant as determined by an ANOVA (*P < 0.05*). *SA* WT, *S. aureus* RN6390; *PA* WT, *P. aeruginosa* PA14. *norA* and *tet38* transcripts of *SA* WT exposed to *PA* QS molecules. *SA* WT (10^5^ CFU/mL) was exposed to *PA* WT supernatant, HHQ, PQS, and PYO at 0.5 × MIC concentrations (**C**), or to PYO concentrations (0–3 µg/mL) (**D**) for 1 hour, and then *norA* and *tet38* transcripts were determined by qPCR assays. The relative transcript levels of *norA* and *tet38* were expressed as the fold change (FC) in pump gene transcripts of *SA* WT grown in TSB and exposed to *PA* WT supernatant or QS molecules versus non-exposed *SA* WT. The assays were repeated three times with three different biological samples. The error bars represent the means of FC ± SEM for each assay. The (*) in (**C**) represents the differences in the FC of *tet38* of *SA* WT exposed to *PA* WT supernatant or QS molecules versus *SA* WT non-exposed. The (**) in (**C**) represents the differences in the FC of *norA* or *tet38* of *SA* WT exposed to QS molecules versus *SA* WT exposed to *PA* WT supernatant. The (*) in (**D**) represents the differences in the FC of *norA* of *SA* WT exposed to PYO at 0.25 µg/mL versus *SA* WT non-exposed (0 µg/mL). The differences were statistically significant as determined by an ANOVA (*P < 0.05*). *SA* WT, *S. aureus* RN6390; *PA* WT, *P. aeruginosa* PA14.

Compared to *SA* WT in TSB, exposure to the supernatant of *PA* WT increased *norA* and *tet38* of *SA* WT twofold and fourfold, respectively (Fig. 3A). To evaluate the clinical relevance of these effects of *PA* supernatants, we also tested the supernatants of four *PA* clinical isolates from patients with co-infection with *SA* and *PA* (PA-12, PA-13, PA-14, and PA-15) (Tables 1 and 3). Exposure of *SA* WT to supernatants of clinical CF-*PA* (PA-12–PA-15) increased *tet38* expression approximately threefold but showed no significant increase in *norA* expression (Fig. 3A).

Compared to *SA* WT in ASM, exposure to the supernatant of *PA* WT and the four CF-*PA* (PA-12–PA-15) decreased *SA* WT *norA* transcript ≤twofold and increased *tet38* 4.5-fold to fivefold (Fig. 3B). Thus, exoproducts of CF clinical isolates also induce expression of *tet38*.

To assess the contributions of specific *PA* exoproducts to the induction of Tet38 and NorA seen with *PA* supernatants, we tested PQS, HHQ, and PYO. We determined the MICs of *PA* molecules and carried out the same exposure assays as were done with *PA* WT supernatant, but using 0.5× MIC of these *PA* molecules. *SA* WT (10^5^ CFU/mL) in TSB was exposed to PQS at 2.5 µg/mL (MIC = 5 µg/mL), HHQ at 80 µg/mL (MIC = 160 µg/mL), and PYO at 3 µg/mL (MIC = 6 µg/mL) for 1 hour at 37 °C under shaking,. ThenqPCRs were performed to compare the *norA* and *tet38* transcript levels under various conditions.

Compared to *SA* WT without exposure, PQS (0.5× MIC) induced the expression of *tet38* fourfold but had minimal effect on *norA*; HHQ and PYO (both at 0.5× MIC) had minimal effects on both pump transcript levels (Fig. 3C). As previously reported (24), PYO induced the expression of *norA* twofold at 0.25 µg/mL (0.04× MIC) but not at 3 µg/mL (0.5× MIC) (Fig. 3D). Compared to *SA* WT in coculture with *PA* WT, PQS (0.5× MIC) induced the expression of *tet38* 1.5-fold but reduced *norA* twofold. This increase in *tet38* suggested a compensation phenomenon. HHQ (0.5× MIC) reduced both *norA* (twofold decrease) and *tet38* (2.5-fold decrease) transcript levels; PYO (0.5× MIC) had minimal effects on both pumps (Fig. 3C).

Taken together, these data indicated that *SA* responds to specific components of the ASM medium (mucin, extracellular DNA) and *PA* QS-secreted small molecules (PQS, HHQ, and PYO) to affect the expression of NorA and Tet38, with the response to *PA* exotoxins possibly reinforcing the adaptation of *SA* to persistence under conditions of the CF airway as in ASM.

Notably, PQS at 0.5× MIC reduced NorA, but Tet38 compensated for its reduction. This likely means that PQS has no significant effect on efflux.

### Efflux pump overexpression is prevalent in *S. aureus* isolates from pwCF

To evaluate the clinical relevance of the findings, we also evaluated *SA* clinical isolates from pwCF. We have collected *SA* clinical strains (CF-*SA*) from sputum and throat swabs of pwCF at the MGH Cystic Fibrosis Center for a period of 21 months from February 2023 to November 2024. The majority of pwCF (range from 2 to 65 years of age) were on highly effective CFTR modulator therapy (*n* = 70, 78%). We randomly selected 20 CF-*SA* (MSSA or MRSA) isolated alone or with other bacteria, and 10 CF-*SA* co-isolated with *P. aeruginosa* (CF-*PA*) to form a sub-set of 30 strains and conducted MIC testing. *spa* typing of these isolates indicated that they were all non-clonal, distinct strain types (data not shown). *S. aureus* Newman, a MSSA strain frequently used as a reference for clinical strains, was included (WT) in the MIC determination (39). Out of 30 CF-*SA*, 9 strains showed an increase ranging from 2-fold to 256-fold or more in the MIC of ciprofloxacin (CIP, 1 µg/mL to >128 µg/mL), and 5 strains showed an increase of fourfold in the MIC of tetracycline (TET) compared to the MICs of *S. aureus* WT (0.5 µg/mL for CIP and TET) (Table 1). Commonly, mutations associated with CIP resistance, such as mutations in the subunits of DNA gyrase (*gyrA*) or topoisomerase IV (*parC*), lead to a high MIC of CIP (40, 41). To evaluate the effect of overexpression of NorA on the CIP MICs of CF-*SA* strains, we determined their MICs of pyocyanin (PYO), also a substrate of the NorA efflux pump (24). Compared to the MICs of *S. aureus* WT (MIC of CIP = 0.5 µg/mL; MIC of PYO = 6 µg/mL), we found that 9 CF-*SA* strains (CF-1, CF-23–CF-30) had an increase in MICs of CIP of 16-fold or more (MICs ~8 to ≥128 µg/mL) that also showed an increase of twofold to fourfold in the MICs of PYO (MICs ~12–24 µg/mL) (Table 1), which is a phenotype associated with NorA overexpression (24). These MICs of PYO suggested that CF-*SA* with a fourfold increase in MIC of CIP exhibited an overexpression of the NorA efflux pump, and CF-*SA* with higher MICs of CIP carried more than one type of mutation. CF-*SA* strains (CF-1, CF-5, CF-23, CF-24, and CF-30) also showed a fourfold increase in the MICs of TET compared to that of the WT strain (MIC of TET = 0.5 µg/mL). These MIC values are similar to the resistance phenotype associated with a Tet38 efflux pump overexpression (42) (Table 1).

We then performed quantitative real-time reverse transcription-PCR (qPCR) to evaluate the overexpression pattern of *norA*, *tet38*, and genes for other clinically relevant efflux pumps such as *norB*, *norC, mepA*, *mdeA*, and *abcA* (22, 24, 32, 43–47) of the 30 clinical strains and compared the results to those of the *SA* WT. CF-*SA* and WT were grown in TSB until mid-log phase (OD_600_ ~0.5), and then RNA was extracted for real-time qPCR. The housekeeping gene *gmk* was used as an internal control for the qPCR assay.

Compared with WT, we found that 14 CF-*SA* of the 30 randomly selected *S. aureus* strains (46.7%) had overexpression of threefold or more in the transcript levels of at least one efflux pump. Most notably, the 9 CF-*SA* strains with an increase in MICs of CIP and PYO out of the 14 pump-overexpressors (64%) also showed an increase of threefold or more in the *norA* transcript levels. Five CF-*SA* out of the 14 pump-overexpressors (35.7%) showed an increase of ~threefold in the *tet38* transcript. Among these 30 strains, 4 CF-*SA* (CF-1, CF-23, CF-24, and CF-30) showed an increase in both *norA* and *tet38* (13.3%) (Table 2). We also found three CF-*SA* with a fourfold increase in *norB* (10%), two CF-*SA* with a fourfold increase in *mepA* (6%), and two CF-*SA* with a threefold increase in *mdeA* (6%) (data not shown). No strains had an increase in transcript levels of *norC* or *abcA*.

These data highlight the frequent overexpression of genes encoding efflux pumps in *S. aureus* isolates infecting pwCF, particularly *norA* and *tet38*.

### Expression of efflux pumps in CF clinical isolates also responds to growth conditions similar to those in CF sputum

To examine the effects of the CF environment on *norA* and *tet38* efflux pump expressions, we cultured 16 CF-*SA* with a wild-type *norA* promoter (CF-1–CF-16) and *SA* Newman (*SA* WT) in ASM medium (14) and carried out qPCR assays to compare the *norA* and *tet38* transcripts of CF-*SA* grown in ASM versus in TSB (Tables 1 and 2). The housekeeping gene *gmk* was used as an internal control, and the *gmk* transcript level remained unchanged whether *SA* was cultured in TSB or ASM. We performed DNA sequencing and found no mutation in the *norA, norB, mdeA*, and *mepA* (fluoroquinolones resistance efflux pumps) or *tet38* (tetracycline resistance efflux pump) of the 16 CF-*SA* with a wild-type *norA* promoter.

**Fig 4:**
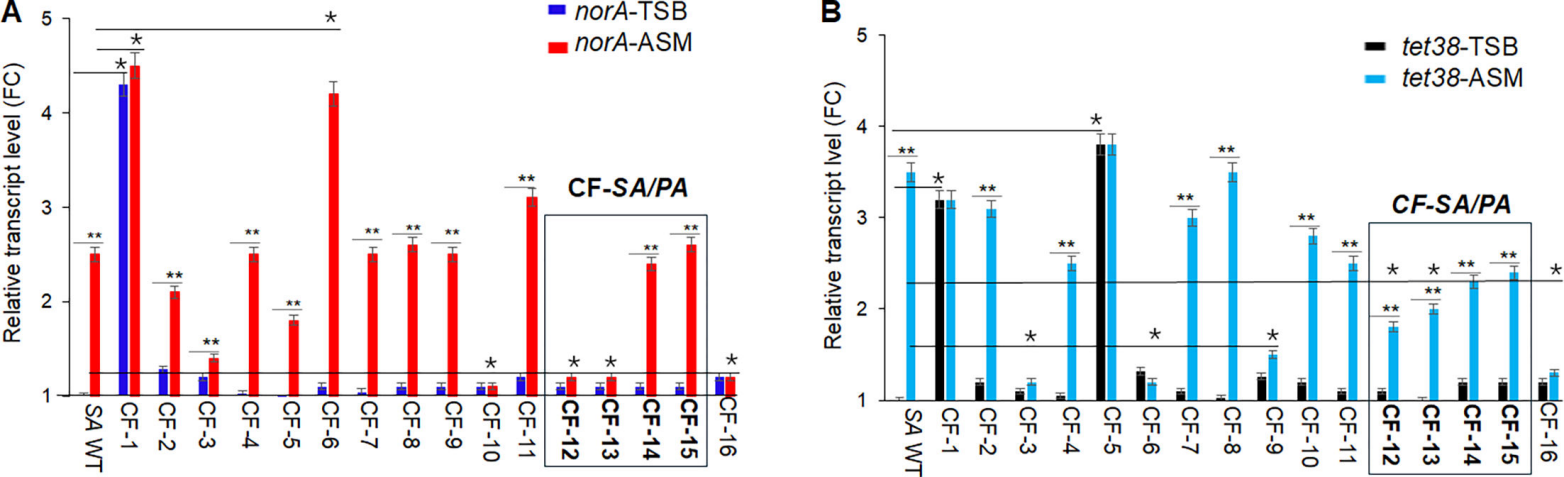
CF-*SA norA* and *tet38* transcript levels and roles of efflux pumps in *S. aureus* ability to grow in ASM medium. 16 CF-*SA* with wild-type *norA* and *tet38* promoters were cultured in TSB and ASM media (initial CFU/mL ~ 10^4^) for 1 hour, then quantitative real-time RT-PCR assays (qPCR) were performed to assess the level of *norA* (A) and *tet38* (B) transcripts. The relative transcript levels of *norA* and *tet38* were expressed as the fold change (FC) in pump gene transcripts of bacteria grown in ASM versus grown in TSB. The assays were repeated three times with three different biological samples. The error bars represent the means of FC ±SEM for each assay. The (*) represents the differences in the FC of *norA* or *tet38* of WT versus *norA* or *tet38* of CF-*SA*. The (**) represents the differences in the FC of *norA* or *tet38* of *S. aureus* in ASM versus *norA* or *tet38* of *S. aureus* in TSB. The differences were statistically significant as determined by an ANOVA (*P < 0.05*). (A) *norA* transcript level; (B) *tet38* transcript level; CF-*SA/PA*, co-isolated pairs; WT, *S. aureus* Newman.

We found that the *SA* WT strain grown in ASM media showed an increase of 2.5-fold and 3.5-fold in *norA* and *tet38* transcript levels, respectively, when compared to that of *SA* WT grown in TSB (Fig. 4A and B). Out of 16 CF-*SA* with wild-type *norA* and *tet38* promoters cultured in ASM, 10 strains showed an increase between 2.1 and 4.2-fold in *norA* (Fig. 4A) or *tet38* (Fig. 4B) compared to that of the same CF-*SA* strains in TSB. Thus, ASM induction of expression of these efflux pumps is also seen in the majority of CF clinical isolates. Notably, in ASM medium, the four CF-*SA* isolated from patients with *PA* co-infection overexpressed *norA* and *tet38* differently. CF-12, CF-13, CF-14, and CF-15 all overexpressed *tet38*, but only CF-14 and CF-15 also overexpressed *norA*. These data suggested that the expression of NorA and Tet38 in CF *SA* may depend on the presence and the behavior of the co-isolated *PA* in a CF environment.

### Distinct *norA* promoter mutations are enriched in CF clinical isolates

We examined the *norA* and *tet38* structural genes and promoter regions of CF-*SA* by comparing *norA* and *tet38* of the published genome of *S. aureus* NCTC8325 (42, 48, 49) with the DNA sequences of CF-*SA* efflux pump genes. No mutation was found in the *tet38* promoter regions or structural genes. We also found no mutation in the *norA* structural genes but identified three types of *norA* promoters among the 30 CF-*SA*: 16 CF-*SA* carried a wild-type *norA* promoter; 6 CF-*SA* carried an insertion of 341 bp of the *S. aureus polX* gene at the (−10) sequence of the *norA* promoter; and 8 CF-*SA* carried a 4-nucleotide insertion (CAAT, ACAA, or CTAT) directly at the (−10) consensus sequence of the *norA* promoter. The insertion of the 341 bp of the *SA polX* gene (encoding a DNA polymerase) disrupted the promoter region that led to a decrease of two- to fivefold in the *norA* transcripts of CF-*SA* when compared to that of WT cultured in TSB (Table 2). By contrast, the *norA* transcripts of the 8 CF-*SA* with a 4-nucleotide promoter insertion showed an increase of 6-fold to ~15-fold when compared to that of WT when cultured in TSB (and a similar increase in ASM). We found 5 out of 10 CF-*SA* of the CF-*SA*/CF-*PA* co-infection pairs carried the insertions CAAT, ACAA, or CTAT (CF-23, CF-27, CF-28, CF-29, CF-30) (ratio 50%) (Tables 1 and 2).

The CAAT insertion was previously reported to cause an increase in *norA* transcripts and the MICs of NorA substrates of other *S. aureus* clinical isolates (45, 50). To confirm the effects of the 4-nucleotide insertion mutations, we generated plasmid constructs carrying the *norA* wild-type gene fused to the *norA* promoter (wild type or with CAAT, ACAA, or CTAT insertion). Plasmid constructs were introduced into *SA* WT (RN6390) and mutant *∆norA*, then the transformants were cultured in TSB or ASM for 1 hour at 37 ºC under shaking, for real-time qPCR assays to evaluate the *norA* transcript levels. We also performed MIC testing to evaluate the level of resistance to CIP and PYO of the transformants (Tables 1 and 3).

**Fig 5:**
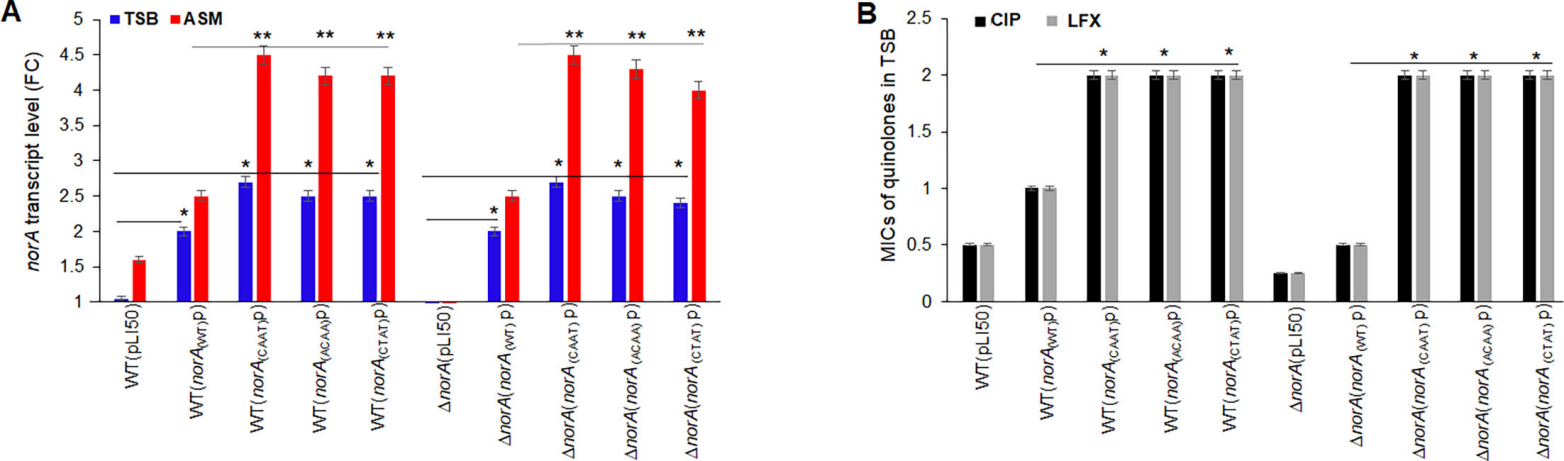
Relative *norA* transcript levels and MICs of ciprofloxacin and levofloxacin of *SA* transformants carrying plasmid-borne *norA* promoter mutations. *norA* transcript level of *SA* transformants in ASM versus TSB. *SA* RN6390 (WT) and *norA* mutant (*∆norA*) transformed with plasmid constructs: pLI50-[*norA*_(WT)_p-*norA*], pLI50-[*norA*_(CAAT)_p-*norA*], pLI50-[*norA*_(ACAA)_p-*norA*], and pLI50-[*norA*_(CTAT)_p-*norA*] were cultured in TSB or ASM (initial CFU/m L~10^4^) supplemented with chloramphenicol (Cm = 10 µg/mL, selection pressure) for 1 hour, then qPCRs were performed to assess the *norA* transcript levels of transformants. (**A**) The relative *norA* transcript levels were expressed as the fold change (FC) in *norA* transcripts of transformants grown in ASM versus in TSB, and the fold change between transformants with plasmid constructs carrying wild-type *norA* promoter versus mutated *norA* promoter. The assays were repeated three times with three different biological samples. The error bars represent the means of FC ± SEM for each assay. The (*) represents the differences in the *norA* transcript levels of WT or *∆norA* mutant with plasmid constructs (WT or mutated *norA* promoters) versus WT or *∆norA* mutant with empty plasmid (pLI50) in TSB. The (**) represents the differences in the *norA* transcript levels of WT or *∆norA* mutant with plasmid constructs (mutated *norA* promoters) versus WT or *∆norA* mutant with plasmid construct pLI50-[*norA*_(WT)_p-*norA*] in ASM. The differences were statistically significant as determined by an ANOVA (*P < 0.05*). MICs of ciprofloxacin and levofloxacin of *SA* transformants. The MICs were carried out in TSB media. (**B**) The (*) represents the differences between the MICs of CIP or LFX between transformants with a *norA* wildtype versus a mutated *norA* promoter. The differences were statistically significant as determined by an ANOVA (*P < 0.05*). WT or *∆norA* (*norA*_(WT)_p), *S. aureus* with construct pLI50-*norA*_(WT)_p-*norA*. WT or *∆norA* (*norA*_(CAAT)_p), *S. aureus* with construct pLI50-*norA*_(CAAT)_p-*norA*. WT or *∆norA* (*norA*_(ACAA)_p), *S. aureus* with construct pLI50-*norA*_(ACAA)_p-*norA*. WT or *∆norA* (*norA*_(CTAT)_p), and *S. aureus* with construct pLI50-*norA*_(CTAT)_p-*norA.*

In TSB, the *norA* relative transcript levels of transformant *SA* WT[pLI50-*norA*_(WT)_p-*norA*) showed an increase of twofold, while transformants *SA* WT[pLI50-*norA*_(CAAT)_p-*norA*), *SA* WT[pLI50-*norA*_(ACAA)_p-*norA*), and *SA* WT[pLI50-*norA*_(CTAT)_p-*norA*) showed an increase of ~2.6 fold when compared to the *norA* transcript levels of *SA* WT (pLI50) (Fig. 5A).

In ASM, the *norA* relative transcripts of transformants *SA* WT (pLI50) and *SA* WT[pLI50-*norA*_(WT)_p-*norA*) showed an increase of 1.6-fold and 2.5-fold, respectively, when compared to the *norA* transcript of bacteria in TSB. Transformants *SA* WT[pLI50-*norA*_(CAAT)_p-*norA*), *SA* WT[pLI50-*norA*_(ACAA)_p-*norA*), and *SA* WT[pLI50-*norA*_(CTAT)_p-*norA*) showed 4.2-fold to 4.5-fold aincrease when compared to the *norA* transcript levels of *SA* WT (pLI50) in TSB.

Thus, in ASM medium, the *norA* transcripts of CAAT/ACAA/CTAT transformants showed ~twofold additional increase in *norA* transcripts when compared to those of transformants with a WT *norA* promoter (Fig. 5A).

We repeated the assay with mutant *∆norA* transformed with the same plasmid constructs. No *norA* transcript was detected in *∆norA* (pLI50 empty plasmid), and we obtained a similar increase in *norA* transcript of *∆norA* transformants as was observed with *SA* WT transformants (Fig. 5A).

We confirmed the *S. aureus* resistance to quinolones phenotype of the transformants by MIC testing. Compared to the MICs of CIP and levofloxacin (LFX) of *SA* WT(pLI50) and *∆norA*(pLI50), *SA* WT[pLI50-*norA*_(WT)_p-*norA*) and *∆norA* [pLI50-*norA*_(WT)_p-*norA*) showed a twofold increase while *S,A* WT and *∆norA* with the CAAT/ACAA/CTAT *norA* promoter showed a fourfold increase in their respective MICs (Fig. 5B).

Compared to the MICs of *SA* WT(pLI50) (CIP, 0.5 µg/mL; PYO, 6 µg/mL), we found an increase of twofold in the MICs of CIP and PYO of *SA* WT[pLI50-*norA*_(WT)_p-*norA*) (1 and 12 µg/mL, respectively) while *SA* WT transformed with plasmid constructs carrying *norA* promoter with insertion CAAT, ACAA, and CTAT, shwed a fourfold increase in MIC of CIP and threefold to fourfold increase in MIC of PYO (Table 1). We found a similar increase in MICs of CIP and PYO of *∆norA* transformants as was observed with *SA* WT, but *∆norA*(pLI50) and *∆norA* [pLI50-*norA*_(WT)_p-*norA*) MICs were twofold less compared to that of *SA* WT transformed with the same plasmids (Table 1). MICs of TET of transformants remained unchanged and similar to that of *SA* WT(pLI50) (TET, 0.5 µg/mL).

To assess the frequency of the CAAT/ACAA/CTAT insertion in a larger subset of CF-*SA*, we performed DNA sequencing of the *norA* promoter region of 18 additional CF-*SA* that were isolated alone or with other bacteria from the same collection. For a total of 48 CF-*SA* (30 + 18 additional strains), we found 3 CF-*SA* out of 38 (in the total 48 CF-*SA* strains, 38 CF-*SA* were isolated alone or with other bacteria, and 10 CF-*SA* were co-isolated with *PA*) that showed a CAAT insertion in their *norA* promoter (3/38 CF-*SA*; 7.9%). This rate was similar to the value observed by a previous study of *S. aureus* bloodstream clinical isolates (~8%) (45). Notably, the rate of the 4-nucleotide insertions was higher among CF-*SA* co-isolated with CF-*PA* (5/10 CF-*SA/PA*, 50%) compared to CF-*SA* isolated alone or with other bacteria (7.9%). In total, the majority of 48 CF-*SA* showed a wild-type *norA* promoter (50%) or a *polX* insertion (33.3%), and 16.7% of CF*-SA* had a 4-nucleotide insertion (CAAT/ACAA/CTAT).

### Effect of mutations causing NorA overexpression on growth in the CF airway environment

**Fig 6:**
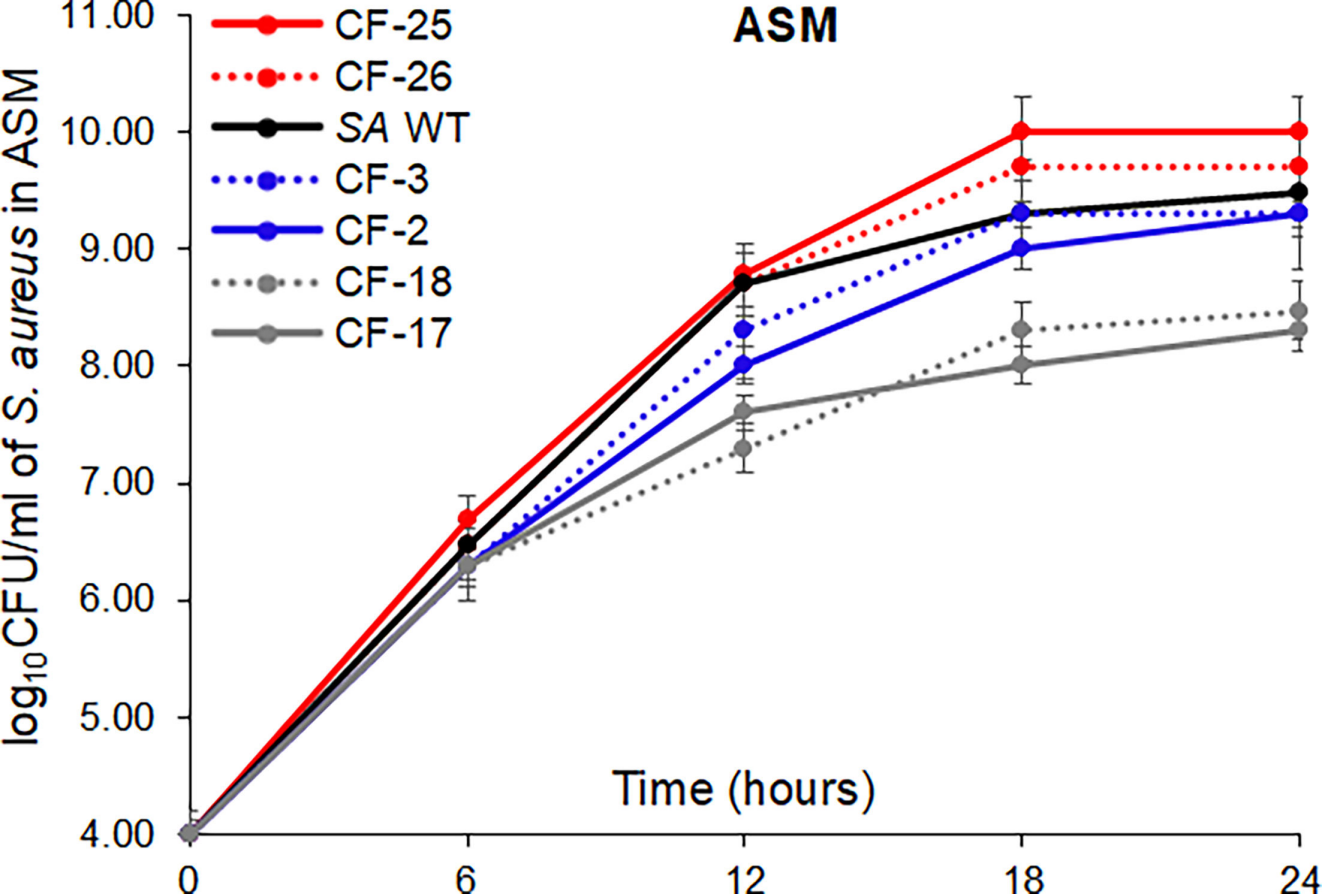
Growth of *SA* in ASM medium. *SA* WT (RN6390), CF-*SA* with CAAT insertion (CF-25, CF-26), CF-*SA* with wild-type promoter (CF-2, CF-3), and CF-*SA* with *polX* insertion (CF-17, CF-18) (initial CFU/mL ~ 10^4^) were cultured in ASM medium for 24 hours, and then colony counting was performed. The growth curve assays were repeated three times with three different biological samples. The error bars represent the means of (log_10_CFU/mL ± SD) for each assay. The differences in the log_10_CFU/mL of CF-17 and CF-18 (*polX* insertion) compared to that of *SA* WT and other CF-*SA* with wildtype or CAAT insertion at 24 hours of growth were statistically significant as determined by an ANOVA (*P <* 0.05).

To determine whether the 4-nucleotide insertion of the *norA* promoter affected *SA* growth in CF sputum conditions in CF clinical isolates, we performed growth curve assays using *SA* WT (RN6390), 2 CF-*SA* with wildtype *norA* promoter (CF-2, CF-3), 2 CF-*SA* with the CAAT insertion mutations (CF-25, CF-26), and 2 CF-*SA* with *polX* insertion (CF-17, CF-18) (Table 1). The *norA* and *tet38* transcript levels of CF-2 and CF-3 were similar to those of *SA* WT; CF-25 and CF-26 showed a sixfold increase in the *norA* transcripts but a similar *tet38* transcript level to those of *SA* WT; CF-17 and CF-18 showed a decrease of fivefold in *norA* but no change in *tet38* (Table 2). We started fresh cultures of *SA* WT and CF-*SA*s in ASM from overnight cultures in TSB with a starting CFU/mL of each strain ~10^4^. After 24 hours, we found that CF-*SA* with CAAT mutation (CF-25 and CF-26) and CF-*SA* with wild-type *norA* promoter (CF-2 and CF-3) exhibited a similar growth rate to that of *SA* WT. Notably, CF-*SA* with a *polX* insertion (CF-17 and CF-18) showed a 1− log_10_CFU/mL growth deficit when compared to the growth rates of other strains, which might be due to a decrease in NorA expression (Table 2 and Fig. 6).

## DISCUSSION

Antibiotic resistance due to overexpression of *S. aureus* (*SA*) efflux pumps has been extensively investigated (22, 51). Recently, our studies have identified additional functions of NorA and Tet38 efflux pumps that include the transport of many compounds in addition to antibiotics, and that some compounds can act as both substrates and inducers of *SA* efflux pumps (24, 25). This study addressed the roles of NorA and Tet38 in *SA* survival in the nutrient-scarce CF environment, alone or in coinfection with *P. aeruginosa* (*PA*). We observed the consequences of this adaptation on *SA*’s ability to decrease susceptibility to antibiotic substrates of NorA and Tet38, to resist killing by *PA* exotoxins, and to persist in the airways of pwCF.

Notably, both NorA and Tet38 had key roles in the ability of *SA* to grow under conditions mimicking those in the CF airways using the artificial sputum ASM, dependencies not seen in laboratory media such as TSB. Growth curve assays showed that a lack of NorA significantly impaired bacterial survival, despite the presence of Tet38 in a *ΔnorA* mutant. By contrast, a lack of Tet38 reduced *SA* growth in ASM, but the effect was less severe, due to the presence of NorA in a *Δtet38* mutant. Furthermore, we found that two key components of the ASM medium, mucin and extracellular DNA, affected the expression of NorA and Tet38 in a complementary manner; mucin repressed *tet38* but activated *norA*, while DNA activated *tet38* but repressed *norA*. Interestingly, the *ΔnorA* mutant survived better in the absence of mucin (porcine stomach), compared to its survival in the absence of DNA (fish sperm) in ASM medium, probably due to the inhibitory effect of mucin on Tet38. By contrast, the *Δtet38* mutant had growth difficulty in the absence of mucin despite the presence of an intact NorA, possibly due to the inhibitory effect of DNA on NorA. These data indicated that Tet38 as well as NorA was important for *SA* growth under conditions simulating CF sputum.

Many studies have investigated the complexities of *SA* and *PA* co-infections, including studies that describe how *PA* pushes *SA* toward a fermentative metabolism, which eventually is detrimental, and how coinfection with *PA* promotes *SA* toward a small-colony-variant (SCV) mode of growth (52, 53). These studies were conducted using various systems, including murine models as well as *in vitro* co-culture in ASM medium (8, 54). However, the activation of *SA* efflux pump production by QS signal molecules during a co-infection of *SA* with *PA* has not been evaluated. Previous studies showed that under the control of MvfR-regulated *PhnAB* and *pqsABCDE* operon, HHQ was synthesized from anthranilic acid (AA) and released from *PA* cells. Then HHQ was taken up by neighboring bacteria to be converted into PQS by the PqsH enzyme controlled by the QS regulator LasR (28, 55). We previously reported that *SA* NorA and Tet38 efflux pumps were induced by their respective substrates PYO (NorA) and PCA (Tet38) of *PA* (24, 25). In this study, we demonstrated that HHQ and PQS, together with PYO, are utilized by *SA* to manage the expression of critical efflux pumps such as NorA and Tet38 in the presence of *PA*. Since HHQ and PYO reduced both NorA and Tet38 expressions, while PQS reduced only NorA, but this phenomenon was compensated by Tet38. This suggested that PQS might have no significant effect on *SA* efflux. Further investigations are underway to elucidate this phenomenon.

We first evaluated *SA* response to *PA* QS secreted products by exposing *SA* to supernatant prepared from a *PA* wild type (30% vol/vol) and compared the *norA* and *tet38* transcript levels of *SA* exposed to *PA* supernatant versus non-exposed. We showed that *PA* WT supernatants containing a mixture of secreted exotoxins and signaling molecules had a higher inducing effect on *tet38* than on *norA* transcripts (fourfold versus twofold increase). In our previous study and again confirmed in this study, we showed that PYO exotoxin at low concentrations (0.04× MIC) induces *norA* expression but not *tet38* (24), suggesting that there is an effect of other *PA* exotoxins or molecules in *PA* supernatants on *tet38* expression, in addition to PCA that we previously reported (25). On exposure to other *PA* QS signal molecules, we found that PQS also induced *tet38* but inhibited *norA*, while HHQ inhibited both *norA* and *tet38*. The concentration of HHQ (80 µg/mL) used was based on the level of HHQ *in vivo* as previously reported by Que et al. (31), and the concentration of PQS (2.5 µg/mL) was based on the level of PQS produced by the *PA* PA14 strain, used as the *PA* WT reference strain in this study, after 18 hours of growth (56). Thus, the effects of exposure to *PA* growth supernatants on *SA* efflux pump expression likely result from the complex interactions of multiple signaling molecules and exotoxins with *SA* that also affect its adaptations to growth in conditions reflecting the milieu of CF sputum.

Taken together, our study using pump mutants clearly demonstrated that *SA* uses NorA and Tet38 to adapt to a nutrient-limited milieu such as the ASM medium as well as to respond to the presence of *PA*. To determine whether these types of *SA* adaptations also occurred in *SA* isolates infecting CF patients, we randomly selected a sub-set of 48 CF-*SA* isolates from a collection of ~70 clinical strains isolated from pwCF. Among these strains, 18.8% CF-*SA* overexpressed *norA* (9/48 strains) and 10.4% overexpressed *tet38* (5/48 strains) relative to a laboratory reference strain. The NorA-overexpressors showed an increase of ≥16 fold in the MIC of ciprofloxacin, two- to fourfold in the MIC of PYO, and the Tet38-overexpressors showed a fourfold increase in the MIC of tetracycline. The NorA-overexpressors with an increase in MIC of ciprofloxacin exceeding fourfold likely also carried additional mutations such as those in quinolone targets DNA gyrase or topoisomerase IV (57). Compared to the *norA* gene of *SA* NCTC8325 or Newman (49, 58), we found that 8 CF-*SA* out of 9 NorA-overexpressors showed an insertion of a 4-nucleotide (CAAT, ACAA, or CTAT) at the −10 motif of the *norA* promoter. Furthermore, 50% of CF-*SA*/*PA* pairs (5/10 strains) carried the 4-nucleotide insertion.

In comparison of our collection of clinical *SA* isolates from pwCF to previously reported other collections of *SA* clinical isolates, the proportion of CF-*SA* with 4-nucleotide *norA* promoter insertions in pwCF (8 CF-*SA* with CAAT/ACAA/CTAT out of 48 isolates) to the proportion of these insertions in other previously reported collections of clinical *SA* isolates, the occurrence was higher (8 CF-*SA* with CAAT/ACAA/CTAT out of 48 isolates, 16.7%) than in a collection of bloodstream isolates (19 *SA* with CAAT/AAT out of 232 clinical *SA*, 8.2%) (45) (*P* = 0.04, Fisher’s exact test), or a collection of hospital-associated isolates (no *SA* with *norA* insertion out of 115 clinical *SA*) (43) (*P* < 0.001, Fisher’s exact test). These data suggested there might be an enrichment of the 4-nucleotide *norA* promoter mutations causing *norA* overexpression in *SA* strains from pwCF, highlighting the likely clinical importance of NorA expression in *SA* adaptation to the CF airway environment (43, 45, 47). As no mutation was found in *tet38* of CF-*SA*, the increase in *tet38* transcripts and TET MICs likely resulted from other regulatory mutations, also possibly driven by the CF airway environment.

This study demonstrates the common overexpression of efflux pumps in *SA* infecting pwCF and the influence of key components of the CF sputum, as well as *PA* signal molecules, on the expression of *SA* NorA and Tet38 efflux pumps. These properties may promote *SA* persistence in the airways of pwCF, causing chronic lung coinfections, in addition to an increase in *SA* resistance to quinolones and tetracycline and other substrates of NorA and Tet38. Further investigations were underway to determine the regulatory mechanisms mediating the effects of *PA* signal molecules on *SA* efflux pumps. Findings from the study could provide new therapeutic approaches targeting *SA* and *PA* co-infections.

## MATERIALS AND METHODS

### Bacterial strains, ASM media, and growth conditions

Bacterial strains, plasmids, and primers used in this study are listed in Table 3. Ciprofloxacin, levofloxacin, tetracycline, chloramphenicol, pyocyanin (PYO), 3,4-dihydroxy-2-heptylquinoline (PQS), 4-hydroxy-2-heptylquinoline (HHQ), mucin from porcine stomach, fish sperm DNA, L-amino acids, diethylenetriaminepentaacetic acid (DTPA), NaCl, KCl, and egg yolk emulsion were purchased from Sigma-Aldrich (St. Louis, MO).

*S. aureus* containing plasmid pLI50 and various derived constructs were grown at 37°C under shaking in trypticase soy broth medium (TSB) or artificial sputum medium (ASM) supplemented with chloramphenicol at 10 µg/mL. All other bacteria were grown in TSB medium unless otherwise stated. The ASM medium was prepared as previously described by Kirchner et al. (14). ASM without mucin was prepared without 0.5% mucin from porcine stomach (wt/vol), and ASM without DNA was prepared without 0.4% DNA from fish sperm (wt/vol).

### CF clinical isolates collection

*S. aureus* (CF-*SA*) and *P. aeruginosa* (CF-*PA*) from patient sputum and throat samples were collected following participant consent, and assent when appropriate, at the Massachusetts General Hospital (MGH) Cystic Fibrosis Center (Institutional Review Board [IRB] no. 2011P000620) over a period of 21 months, from February 2023 to November 2024. Patient characteristics and CFTR modulator use were extracted from medical records. Isolates were cultivated in Trypticase Soy Broth (TSB), streaked out on TSA agar plates, and grew at 37 °C for 24 hours. All strains grew normally within 24 hours.

### Antibiotic susceptibility assay

The MIC was determined by broth microdilution at 37°C for 24 hours, as previously described (42). A log-phase culture of *S. aureus* (OD_600_ = 0.5) grown in TSB media was diluted 100-fold and inoculated into microtiter plates (Fisher Scientific, Pittsburgh, PA) containing twofold serial dilutions of ciprofloxacin, levofloxacin, tetracycline, or *P. aeruginosa* signal molecules (PQS, HHQ, and PYO). MIC was the lowest drug concentration that produced no visible turbidity after incubation at 37°C for 24 hours.

### Construction of plasmids pLI50-*norA_WT_*p-*norA* and pLI50-*norA*_(CAAT/ACAA/CTAT)_p-*norA*

We amplified a DNA fragment that carried the *norA* gene together with its promoter (*norA* promoter ~359 bp upstream of the *norA* gene) from *S. aureus* RN6390 using primers *norA*-PF (*BamH*I) and *norA-EcoR*I-R (Table 3). The primers were designed based on the DNA sequence of the published genome of *S. aureus* NCTC8325 (58). The PCR product and the plasmid pLI50 were digested with restriction enzymes *BamH*I and *EcoR*I, then ligated to generate the plasmid construct pLI50-*norA*_WT_p-*norA*. This technique was previously described in our other studies (25, 39). We replaced the *norA* promoter of construct pLI50-*norA*_WT_p-*norA* using primers *norA*-P-F1, *norA*-P-F2, and *norA*-P-F3 as forward primers and *norA*-P-R as reverse primer (Table 3) to amplify new *norA* promoters with the insertion mutations CAAT, ACAA, or CTAT. The replacements of the *norA* wild-type promoter (*norA*_WT_p) by the mutated *norA* promoter were carried out using the plasmid construct pLI50-*norA*_WT_p-*norA* as template and the Phusion Site-Directed Mutagenesis Kit (Fisher Scientific, Pittsburgh, PA) as recommended by the manufacturer, to generate new plasmid constructs pLI50-*norA*_CAAT_p-*norA*, pLI50-*norA*_ACAA_p-*norA*, and pLI50-*norA*_CTAT_p-*norA*. The plasmid constructs were introduced into *S. aureus* RN6390 and *∆norA* mutant, and transformants were selected on TSB agar plates supplemented with chloramphenicol 10 µg/mL.

### Quantitative real-time RT-PCR assay

The real-time RT-PCR assays were done as previously described (24). Total *S. aureus* RNA was extracted from lysostaphin-treated cells using the RNeasy midi kit (Qiagen, Valencia, CA). cDNAs were synthesized using the Verso cDNA synthesis kit (Thermo Scientific, ABgene, Epsom, Surrey, United Kingdom), followed by real-time RT-PCR assays using EvaGreen dye and the CFX96 real-time system (Bio-Rad, Hercules, CA). Primers designed for the RT-PCR assays were synthesized at Eton Bioscience Inc. (Eton Bioscience, Boston, MA) and are listed in Table 3. The housekeeping gene *gmk* was used as an internal control. All samples were analyzed in triplicate, and expression levels normalized against *gmk* gene expression, which remained unchanged following exposure to ASM media or to 0.5 × MIC concentration of signal molecules or to supernatants of *P. aeruginosa*. The assays were repeated with three independent biological samples. Statistical analyses were performed using a one-way ANOVA with a post hoc *t*-test.

### Supernatant exposure assays

We carried out the supernatant exposure assay as previously described with some modifications (25). We grew *P. aeruginosa* PA14 in TSB media overnight, then followed the protocol described by Niggli et al. (18) to prepare the assay media with a proportion of 30% *P. aeruginosa* supernatant in TSB media. In brief, we centrifuged 10 mL of overnight bacterial cultures and then discarded the pellets and filtered the supernatants using 0.2 µm filter cups. To carry out the *S. aureus* exposure assay, the sterile supernatants were added to TSB or ASM in the proportion of 30% *P. aeruginosa* supernatant + 70% TSB or ASM. In parallel, we grew *S. aureus* RN6390 in TSB media from an overnight culture until OD._600_ reached 0.5. We centrifuged a series of 2 mL of RN6390 and resuspended the pellet in 2 mL of the assay media prepared *P. aeruginosa* PA14 in TSB or in ASM. The supernatant exposure assay was carried out for 1 hour an,d then total RNAs of RN6390 were extracted and submitted to real-time qPCR to determine the relative pump gene *norA* and *tet38* transcript levels of *S. aureus* RN6390. The relative gene transcripts were expressed as the fold change (FC) of RN6390 pump gene exposed versus non-exposed to *PA* supernatants. RN6390 grown in 100% TSB served as a positive control of *S. aureus* growth, and *S. aureus* grew in saline (0.9% NaCl solution) TS.B (30% saline +70% TSB) served as a growth in reduced nutrient control (negative control). We used the gene *gmk* as an internal control of the assay.

### PQS, HHQ, and PYO exposure assays

We determined the MICs of PQS, HHQ, and PYO of RN6390 and carried out the exposure assays using 0.5× MIC concentrations of *P. aeruginosa* molecules. We used a higher concentration of HHQ (80 µg/mL; MIC = 160 µg/mL) than PQS (2.5 µg/mL; 5 µg/mL) or PYO (3 µg/mL; 3 µg/mL) due to a high level of HHQ *in vivo* as previously reported by Que et al. (31). The PQS concentration was determined based on the level of PQS produced by the PA14 strain after 18 hours of growth (5–10 µg/mL) (56). RN6390 grew in TSB until OD_600_ ~0.5 then 0.5×MIC of PQS, HHQ, or PYO was added to separate RN6390 cultures in TSB for 1 hour at 37 °C under shaking. Bacterial pellets were harvested and submitted to qPCR assays to assess the transcript levels of *norA* and *tet38*.

*S. aureus* RN6390 at OD_600_ ~ 0.5 in TSB was exposed to PYO at concentrations ranging from 0 to 3 µg/mL for 1 hour, and then qPCR assays were carried out to determine the transcript levels of *norA* and *tet38*.

### Growth curves of *S. aureus* strains in ASM, ASM without mucin, and ASM without DNA

*S. aureus* RN6390, *ΔnorA* mutant*, Δtet38* mutant, and 16 CF-*SA* with wild-type *norA* and *tet38* genes were cultured overnight at 37°C in TSB media. Then, 10^4^ CFU/mL of each culture was transferred into 10 mL of fresh TSB or ASM media and allowed to grow at 37°C under shaking for a period of 20 hours. Bacterial samples were collected every 2 hours, diluted, and plated on TSB agar for colony counts (CFU/mL) (32).

For qPCR assays, bacteria were collected after 1 hour of growth in TSB or ASM, then RNA was extracted and submitted to real-time RT-PCR assay to evaluate *norA* and *tet38*.

*S. aureus* RN6390, *ΔnorA* mutant*,* and *Δtet38* mutant were cultured overnight at 37°C in TSB media. Then, 10^4^ CFU/mL of each culture was transferred into 10 mL of fresh ASM, ASM without mucin, or ASM without DNA and allowed to grow at 37°C under shaking. All *S. aureus* continued to grow for 24 hours with samples collected every 6 hours for colony counts (CFU/mL). Bacteria were also collected after 1 hour of growth for qPCR assays.

To assess the impact of CAAT mutation on *S. aureus* growth in ASM, we performed curve assays in ASM medium with *S. aureus* RN6390(pLI50-*norA*_WT_p-*norA*), RN6390(pLI50-*norA*_CAAT_p-*norA*), 2 CF-*SA* with CAAT *norA* promoter, 2 CF-*SA* with WT *norA* promoter, and 2 CF-*SA* with *polX* insertion promoter. The growth curve assays were performed as described in this study.

All experiments were repeated using three independent biological samples. Statistical analyses were performed using a one-way ANOVA with a *t*-test as a post hoc test to determine the significance of differences in the growth of *S. aureus* strains RN6390 and pump mutants in different ASM media and compared to growth in TSB.

### Statistical analysis

The experiments were performed in triplicate with three biological samples. The data were expressed as a mean ± SD. The data were analyzed using one-way analysis of variance (ANOVA). The pairwise comparison was done with a *t*-test with Bonferroni adjustment to compare sample groups. The threshold for significance was set at a *P*-value < 0.05.

## SUPPLEMENTAL MATERIAL

Table S1 aac.00460-25-s0001.docx gmk transcript of *S. aureus* cultured in TSB or ASM with or without mucin or DNA.

ASM does not own the copyrights to Supplemental Material that may be linked to, or accessed through, an article. The authors have granted ASM a non-exclusive, world-wide license to publish the Supplemental Material files. Please contact the corresponding author directly for reuse.
