# Supplementary material for: NorA and Tet38 efflux pumps enable Staphylococcus aureus survival in the cystic fibrosis airway environment, resistance to antibiotics, and coinfection with Pseudomonas aeruginosa
Source: Antimicrob Agents Chemother. 2025 Jul 9;69(8):e00460-25. doi: 10.1128/aac.00460-25 (PMC12326990; doi:10.1128/aac.00460-25)
Supplement: Table S1 — gmk transcript of S. aureus cultured in TSB or ASM with or without mucin or DNA. [file aac.00460-25-s0001.docx]

**NorA and Tet38 Efflux Pumps Enable *Staphylococcus aureus* Survival in Cystic Fibrosis Airways Environment, Resistance to Antibiotics, and Coinfection with *Pseudomonas aeruginosa*.**

Truong-Bolduc Q.C., Wang Y., Lawton B.G., Zweifach J. J., McDevitt G. M., El Abdellaoui Y., Brown Harding H., Yonker L.M., Rahme L.G., Vyas J.M., and Hooper D.C.

**Supplementary data**

**Supplementary Data**

**Table S1.** *gmk* transcript of *S. aureus* cultured in TSB or ASM with or without mucin or DNA.

|  |  | Relative transcript level (FC) of *gmk* (mean FC ± SD)* | | | |
| --- | --- | --- | --- | --- | --- |
|  |  |  |  |  |  |
|  |  | TSB | ASM | ASM – Mucin | ASM – DNA |
|  |  |  |  |  |  |
| *S. aureus* |  |  |  |  |  |
|  |  |  |  |  |  |
| RN6390 |  | 1 | 1 | 1 | 1 |
| NCTC8325-4 |  | 1.1 ± 0.01 | 0.9 ± 0.02 | 1.3 ± 0.01 | 1.0 ± 0.02 |
| *∆norA* |  | 1.0 ± 0.01 | 1.1 ± 0.01 | 1.2 ± 0.01 | 1.3 ± 0.02 |
| *∆tet38* |  | 0.9 ± 0.01 | 0.8 ± 0.02 | 1.2 ± 0.02 | 1.1 ± 0.01 |

__________________________________________________________________________

* Relative gene expression determined as the fold change (FC) of *gmk* gene transcript of WT and mutant strains cultured in ASM, (ASM-Mucin), and (ASM-DNA) compared to that of the same strains in TSB. RN6390 serves as a reference for *S. aureus* strains. Each assay was done in triplicate, and RNAs were collected from three independent biological samples. All values represent the means of three independent experiments. The *gmk* transcript level remained unchanged whether *S. aureus* strains were cultured in TSB or ASM with or without mucin or DNA. WT, RN6390 and NCTC8325-4; *∆norA*, *norA* mutant; *∆tet38*, *tet38* mutant; ASM, regular ASM medium; ASM – Mucin, ASM without mucin; ASM – DNA, ASM without DNA.
